# A Model of Germinal Matrix Hemorrhage in Preterm Rat Pups

**DOI:** 10.3389/fncel.2020.535320

**Published:** 2020-12-03

**Authors:** Masako Jinnai, Gabriella Koning, Gagandeep Singh-Mallah, Andrea Jonsdotter, Anna-Lena Leverin, Pernilla Svedin, Syam Nair, Satoru Takeda, Xiaoyang Wang, Carina Mallard, Carl Joakim Ek, Eridan Rocha-Ferreira, Henrik Hagberg

**Affiliations:** ^1^Department of Obstetrics and Gynecology, Centre of Perinatal Medicine, Health, Institute of Clinical Sciences, Institute of Neuroscience and Physiology, Sahlgrenska Academy, Gothenburg University, Gothenburg, Sweden; ^2^Department of Obstetrics and Gynecology, Faculty of Medicine, Juntendo University, Tokyo, Japan; ^3^Henan Key Laboratory of Child Brain Injury, Institute of Neuroscience, Third Affiliated Hospital of Zhengzhou University, Zhengzhou, China

**Keywords:** preterm, brain, germinal matrix hemorrhage, intraventricular hemorrhage, neurodevelopment, neonatal brain

## Abstract

Germinal matrix hemorrhage (GMH) is a serious complication in extremely preterm infants associated with neurological deficits and mortality. The purpose of the present study was to develop and characterize a grade III and IV GMH model in postnatal day 5 (P5) rats, the equivalent of preterm human brain maturation. P5 Wistar rats were exposed to unilateral GMH through intracranial injection into the striatum close to the germinal matrix with 0.1, 0.2, or 0.3 U of collagenase VII. During 10 days following GMH induction, motor functions and body weight were assessed and brain tissue collected at P16. Animals were tested for anxiety, motor coordination and motor asymmetry on P22–26 and P36–40. Using immunohistochemical staining and neuropathological scoring we found that a collagenase dose of 0.3 U induced GMH. Neuropathological assessment revealed that the brain injury in the collagenase group was characterized by dilation of the ipsilateral ventricle combined with mild to severe cellular necrosis as well as mild to moderate atrophy at the levels of striatum and subcortical white matter, and to a lesser extent, hippocampus and cortex. Within 0.5 h post-collagenase injection there was clear bleeding at the site of injury, with progressive increase in iron and infiltration of neutrophils in the first 24 h, together with focal microglia activation. By P16, blood was no longer observed, although significant gray and white matter brain infarction persisted. Astrogliosis was also detected at this time-point. Animals exposed to GMH performed worse than controls in the negative geotaxis test and also opened their eyes with latency compared to control animals. At P40, GMH rats spent more time in the center of open field box and moved at higher speed compared to the controls, and continued to show ipsilateral injury in striatum and subcortical white matter. We have established a P5 rat model of collagenase-induced GMH for the study of preterm brain injury. Our results show that P5 rat pups exposed to GMH develop moderate brain injury affecting both gray and white matter associated with delayed eye opening and abnormal motor functions. These animals develop hyperactivity and show reduced anxiety in the juvenile stage.

## Introduction

Advances and improvement in health care have allowed continual increase in survival of preterm infants, including extremely preterm, i.e., born before gestational week 28. However, this increase in survival is not associated with a consistent reduction in morbidity (Lorenz et al., 1998; Kaiser et al., 2004; Bodeau-Livinec et al., 2008; Seri and Evans, 2008; Maršál et al., 2009; Hinojosa-Rodríguez et al., 2017). Most commonly, preterm infants weighing 500–1,500 g will suffer from neonatal and life-long complications such as respiratory bronchopulmonary dysplasia, a chronic respiratory disease, intestinal necrotizing enterocolitis and germinal matrix hemorrhage (GMH) resulting in intra- and periventricular hemorrhage (IVH/PVH) (Owens, 2005; Kenet et al., 2011).

GMH-IVH is a major cause of preterm brain injury. The germinal matrix (also termed the ganglionic eminence) is only present until gestational week 32 (Whitelaw, 2012). This is a highly vascularized brain area, which is central for development and a major source of neurons and glial cells. The germinal matrix vasculature is fragile, and the combination of reduced cerebral autoregulation and fluctuation of cerebral blood flow can result in vessel rupture within the germinal matrix (Kaiser et al., 2005). This rupture, known as GMH is particularly common in infants born extremely preterm (<28 weeks of gestation) (Kenet et al., 2011).

GMH is divided into four grades, with grades III and IV having the worst outcome (Brouwer et al., 2014). Grade IV cases have a prognosis of 90% mortality, with 80% of survivors suffering from cerebral palsy and cognitive difficulties (Stoll et al., 2004). In these severe cases, GMH results in blood clots which cause cell death as well as impairment in cerebrospinal fluid (CSF) circulation and drainage. There is an excess release of free iron with subsequent free radical production. This normally occurs within the first 72 h of life (Lekic et al., 2015). After resolution of hematoma, there is a secondary wave of tissue loss, as a result of CSF accumulation in the brain (hydrocephalus) causing tissue compression. Re-establishment of blood flow can result in ischemia-reperfusion injury, due to further generation of free radicals and continued oxidative injury, with further damage to various brain cells, particularly oligodendrocyte precursor cells. This induces a strong and prolonged inflammatory response, which further exacerbates white matter damage and results in poor neurological outcome (Brouwer et al., 2014). Around two thirds of severe GMH-IVH cases show significant impairment in both motor and cognitive function (Bassan et al., 2007).

The onset of GMH is difficult to prevent (Roland and Hill, 2003) and current treatment options consist of antenatal administration of corticosteroids and magnesium sulfate (Whitelaw, 2001; Hirtz et al., 2015; Crowther et al., 2017). Postnatally, around 25% of infants with severe GMH-IVH require the insertion of a shunt (Brouwer et al., 2014) and administration of indomethacin has shown a potential benefit (Fowlie and Davis, 2003). Unfortunately, in most cases only supportive care can be provided (Kenet et al., 2011). This has resulted in an urgent unmet need for development of novel treatment strategies. The characterization of standardized animal models for the study of GMH is therefore of great importance if the brain injury and neurological deficits following this condition are to be prevented and/or treated adequately (Ballabh et al., 2007; MacLellan et al., 2008; Chua et al., 2009).

Several animal species such as the mouse, rat, rabbit, sheep and piglet have been used for the study of GMH (Goddard et al., 1980; Balasubramaniam and Del Bigio, 2006; Aquilina et al., 2007; Chua et al., 2009). In these studies, GMH has been induced both via direct methods (blood injected into ventricle) and systemic alterations, including change of hemodynamic properties such as osmolality, oxygenation levels or blood pressure (Goddard et al., 1980; Balasubramaniam and Del Bigio, 2006; Georgiadis et al., 2008). The limitation of these models is that none of them sufficiently resembles the clinical cases. In a study from 2012, Lekic et al. (2011, 2012) reported that infusion of blood into the brain has little relation to a spontaneous bleed and that a hemodynamic property change will lead to secondary injury caused by hypoxia or hypertension. They also mention the lack of rodent models for the study of GMH mimicking the neurological consequences of premature newborns suffering from this condition (Lekic et al., 2011, 2012). In their study GMH is induced by injection of collagenase, a sterile collagen-degrading agent (Rosenberg et al., 1990) in the P7 rat. This causes a standardized, spontaneous rupture of vessels in the germinal matrix of the ganglionic eminence, and extravasation into the lateral ventricles (Lekic et al., 2012), enabling the study of features mimicking those of clinical GMH.

However, more recent studies have shown that P7 rats more closely resembles near-term human infants (Semple et al., 2013), where GMH is not one of the main risk factors for neonatal brain injury. Therefore, the aim of the present study was to develop and characterize a model of severe GMH, i.e., grades III and IV, using P5 rats—corresponding to brain maturation in preterm human infants and at an age when the germinal matrix is still present, and to further characterize the pathophysiology of this disease with the hope of assisting novel experimental therapy studies.

## Materials and Methods

### Surgical Procedure

**FIGURE 1:**
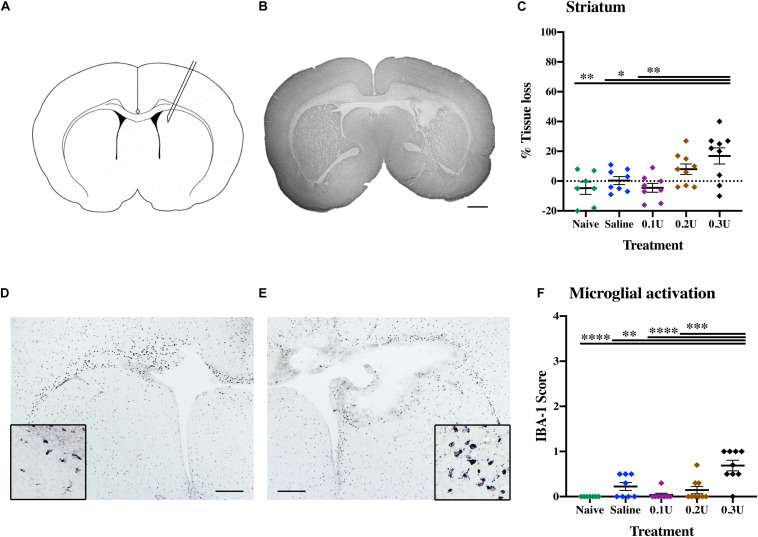
Optimal collagenase dose to induce GMH. **(A)** Diagram of ICV injection site, with needle insertion located at 4 mm lateral of the midline and 1 mm rostral of bregma, with an insertion depth of 3.5 mm. **(B)** Representative MAP-2-stained whole brain micrograph of 0.3 U collagenase-mediated injury 24 h after injection. **(C)** Effect of different treatments—naïve (*n* = 8), saline (*n* = 8), 0.1 U (*n* = 8), 0.2 U (*n* = 9), and 0.3 U (*n* = 9) collagenase—on ipsilateral striatum tissue loss 24 h after injection. **(D)** Contralateral and **(E)** ipsilateral micrographs with magnification inserts (×20) showing IBA-1 microglia staining 24 h after injection. **(F)** Assessment of microglia activation (IBA-1) in the ipsilateral striatum 24 h after different collagenase dose administrations. Data represented as individual animals ± SEM and analyzed using one-way ANOVA followed by Tukey’s multiple comparison test. ^∗^*P* < 0.05, ^∗∗^*P* < 0.01, ^∗∗∗^*P* < 0.001, and ^*⁣*⁣**^P < 0.0001. Scale bar = 1 mm in **(B)**; 0.5 mm in **(D,E)**.

At P5, Wistar rat pups of either sex were randomly allocated into groups and injected into the medial striatum in proximity to the germinal matrix (Figure 1A) with collagenase VII (Sigma-Aldrich, cat# C2399) for induction of GMH or with an equivalent volume of saline as control. Additionally, naïve animals served as needle control. The animals were anaesthetized with isoflurane (5% induction, 1.5% maintenance), and the solution was injected free hand, using a guiding device, into the germinal matrix of the right hemisphere using a 27 G (0.4 mm) needle and a 1 ml Hamilton syringe connected to an infusion pump (CMA/100 microinjection pump). The correct position for needle insertion was located at 4 mm lateral of the midline and 1 mm rostral of bregma and the needle angled medially was inserted to a depth of 3.5 mm and the collagenase was injected with a steady infusion flow of 1 μl/min for 2 min. The needle remained in place for an additional minute following the injection in order to avoid back-flow. After completing the procedure, the animals were allowed to rest on a heating pad set at 35°C and upon recovery from anesthesia all animals were returned to their dams. The duration of the procedure was <5 min/animal. No mortality was recorded in any of the different treatment groups. The animals that survived longer than 24 h were monitored daily for the first 10 days after GMH and no adverse effects were observed. Overall, combining all treatment groups and time-points, a total of 131 animals were used in this study (Supplementary Figure 1).

### Dose Titration

Titration of the optimal collagenase concentration was performed to induce a hemorrhage contained in one hemisphere through infusion into the germinal matrix of the medial striatum (Figure 1A). Based on the study of Lekic et al. (2012), P5 pups were injected with 2 μl of 0.1 U (*n* = 8), 0.2 U (*n* = 9), or 0.3 U (*n* = 9) of collagenase VII (1,000–3,000 CDU/mg solid, C2399, Sigma-Aldrich, Saint Louis, United States) or saline as control (*n* = 8). Naïve animals (*n* = 8) served as needle control. Brains were collected 24 h after injection (P6) and evaluated immunohistochemically as described below.

### Assessment of Early Motor Function

Following collagenase injection (0.3 U, *n* = 12), the development of a subgroup of the pups was blindly assessed over a period of 5 days. *Negative geotaxis* was used to test the amount of time required for a pup to rotate 180° after having been placed head down at a 20° downward slope. Latency of *eye opening* was noted for each eye in all pups. Both naïve (*n* = 10) and saline-injected animals (*n* = 10) were used as control groups.

### Long-Term Sensorimotor and Behavior Tests

Naïve (*n* = 5), saline- (*n* = 12) and collagenase- (*n* = 19) injected animals were used for assessing long-term neurobehavior tests. All animals underwent these tests at two separate time-points, 2–3 weeks (P22–26) and 4–5 weeks (P36–40) after GMH. Brains were collected at P40 for immunohistochemistry.

#### Rotarod

Motor coordination and balance was analyzed using rotarod test on P22 and P36. The apparatus (Panlab, Harvard apparatus) consisted of a horizontal rod (25 cm diameter) with an inter-lane distance of 5 cm (P22) or 7.5 cm (P36). Rats were placed on the rod rotating at a constant speed of 4 rpm. The rotations were accelerated to 40 rpm over a period of 300 s, and the latency to fall was recorded. Each animal underwent three trials, with an inter-trial recovery time of 15–20 min spent in the home cage. The apparatus was cleaned using 70% ethanol between each trial.

#### Cylinder Rearing Test

Motor asymmetry analyzed using cylinder rearing test on P23 and P37. Rats were individually placed in a transparent glass cylinder (14 cm D × 21 cm H and 17 cm D × 28 cm H, for P23 and P37 rats, respectively) and video-recorded for 5 min to analyze forepaw preference during full rearing and lateral exploration as per the criteria suggested by Kadam et al. (2009). Two mirrors (30 × 30 cm) were placed behind the cylinder at an angle such that the forelimb movements could be observed when the rat turned away from the video camera. The forelimb asymmetry was measured by calculating the unimpaired forepaw initiation preference score (%) as follows: (unimpaired–impaired) / (unimpaired+impaired+both) × 100. Animals that made ≤10 full rears were excluded from the study (Schallert et al., 2000).

#### Open Field Test

General locomotor activity and anxiety-like behavior were tested on P23 and P37 using this stress-sensitive behavioral task. The test was conducted in dark for 30 min. To acclimatize the animals to the testing conditions, the animals were brought to the dark anteroom at least 30 min prior to the test. A dark-gray plexiglass square open-top box (100 cm × 100 cm × 40 cm) served as the testing arena. The box was mounted with an infrared-sensitive CCD camera on the ceiling, and two infrared lamps were used to illuminate the arena. The box was virtually divided into a central area (33 cm × 33 cm), corners (15 cm × 15 cm) and a peripheral area (remaining area) using the ANY-Maze tracking software (Stoelting Co.). Mean speed, mobile time and total time spent in each zone was recorded and analyzed using ANY-Maze. ANY-Maze tracked and used the center of animal’s body for recording an entry into a zone. Data was split into 5 min time bins for statistical analysis. The box was cleaned off any feces and urine using 70% ethanol between each trial.

### Tissue Collection and Processing

Brains were collected for histology at five different time-points following ICV injection. One subgroup of the animals was sacrificed 0.5 h (*n* = 7), and 6 h (*n* = 7) after ICV for descriptive assessment of injury progression. P5 naïve animals (*n* = 7) were used as control for both 0.5 and 6 h time-points. A second subgroup of animals were terminated on P6, P16, and P40 for immunohistochemistry and neurobehavioral (P40) measurements. The pups were deeply anesthetized with 0.1 ml of Pentocur and the brains dissected out and immersion fixed in histofix (Histolab Products AB, Sweden). Brains were dehydrated and embedded in paraffin and cut with a microtome into 7 μm thick sections at the level of the striatum (equivalent to adult +0.2 mm from bregma) and hippocampus (equivalent to adult −3.3 mm from bregma) for immunohistochemical staining.

### Histochemistry

Brains were collected at 0.5, 6, and 24 h after GMH surgery for histopathological assessment of injury and bleeding progression. Sections underwent deparaffination in xylene followed by rehydration in graded alcohol. For thionin and acid fuschsin staining, sections were incubated in thionin for 4 min, rinsed in water and then placed in acid fuschsin stain for 30 s. Iron staining was performed using the iron stain kit HT20 (Sigma-Aldrich) according to the manufacturer’s instructions. Perl’s blue staining was further enhanced using DAB peroxidase. All sections were dehydrated in increasing concentrations of alcohol, xylene and covered using Pertex.

### Immunohistochemistry

Brain sections were prepared for immunohistochemical staining by deparaffination in xylene followed by rehydration in graded alcohol. Sections were boiled in 0.01 M citric acid buffer (pH 6.0) for antigen recovery and blocked for endogenous peroxidase (3 % H_2_O_2_) and nonspecific binding (horse and goat serum). Sections were incubated with the primary antibodies: Rabbit anti-glial fibrillary acidic protein (GFAP, Sigma-Aldrich; cat#G3893; 1:400), rabbit anti-ionized calcium binding adaptor molecule 1 (IBA-1, FUJIFILM Wako Chemicals, cat#019-19741; 1:2,000), mouse anti-microtubule-associated protein-2 (MAP-2, Sigma-Aldrich, cat#M4403; 1:1,000), mouse anti-myelin basic protein (MBP SMI-94, BioSite, cat#836504; 1:1,000), rabbit anti-myeloperoxidase (MPO, abcam, cat#AB9535; 1:100) overnight, washed and incubated with an appropriate secondary antibody. ABC elite was used for visualization of immunoreactivity and the sections were submerged into 0.5 mg/ml 3.3-diaminobenzidine (DAB) enhanced with nickel sulfate (15 mg/ml). Sections were dehydrated and mounted as described above.

### Immunofluorescence

Paraffin embedded brains from 24 h survival post-GMH were sectioned at 7 μm, and paraffin removed by xylene, sections hydrated through decreasing concentrations of ethanol and washed in PBS with 0.05% tween20. Antigen retrieval was performed by boiling sections in 0.01 M citrate buffer (pH6.0) for 10 min followed by incubating in serum-free protein blocking solution (Agilent DAKO) for 30 min. Sections were then incubated in a mixture of rabbit anti-laminin antibodies (Novus Biologicals, cat#NB300-144; 1:200) and mouse anti-claudin5 antibodies (Thermo Fisher Scientific, cat#35-2500; 1:200) overnight in fridge. The next day, section were incubated in appropriate alexa-488 and -594 conjugated secondary antibodies for 2 h at room temperature. In between each step above sections were washed in PBS with 0.05% tween20. Finally, due to inherent autofluorescence around injury site, sections were treated using Vector TrueView autofluorescence quenching kit according to manufactures instructions, examined and photographed using an Olympus BX50 microscope fitted with a DP72 camera. All images were processed using Imaris v.9.1 (Bitplane AG).

### Data Analysis

Brain injury was quantified as the area loss of gray matter (MAP-2) and white matter (MBP) immunoreactivity at the levels of striatum and anterior hippocampus in P6 (MAP-2 only), P16 and P40 animals. Total area positive MAP-2 staining in each intact hemisphere and specific brain structures (striatum and hippocampus) and MBP positive staining of the subcortical white matter were outlined and measured using ImageJ (version 1.51, NIH). The percentage of tissue loss was calculated by subtracting the ipsilateral positive area from the corresponding contralateral regions. This method assumes that the contralateral hemisphere represents 100% intact area, however, there are sections, where due to potential symmetrical differences between contralateral and ipsilateral regions, the undamaged ipsilateral hemisphere might be larger, resulting in negative tissue loss. Microglial activation was determined using semi-quantitative score of IBA-1-positive cells, with a scale of 0—no activation, ramified microglia; 1—focal activation, ramified microglia; 2—mild diffusion activation with still predominant ramified microglia; 3—moderate widespread activation with cells showing retraction of the process and swollen cell body; to 4—widespread amoeboid microglia, as previously described (Rocha-Ferreira et al., 2015, 2016). GFAP immunoreactivity was assessed using ImageJ threshold tool. In brief three non-overlapping RGB images from the striatum region were captured at x20 magnification, using an Olympus BX50 microscope fitted with a DP72 camera. Each image was duplicated and transformed into 8-bit grayscale images before the default red threshold setting was applied. The positive red threshold was calculated as percentage of entire image.

### Statistical Analysis

Graph Pad Prism (version 8.3.0) and SPSS were used to perform all statistical analyses. The data was first checked for Gaussian distribution using D’Agostino and Pearson normality test. Kruskal-Wallis Dunn’s test or ANOVA with Tukey’s multiple comparisons test was used to determine statistical significance. The test used for each experiment is stated in the figure legends and *P-*values of < 0.05 were considered to be statistically significant and data is expressed as individual animals or mean ± SEM.

## Results

### Dose Response

To establish the optimal collagenase dose for GMH induction via intracranial injection into the germinal matrix of medial striatum (Figure 1A), a dose response was performed through the injection of three different doses of collagenase (0.1, 0.2, and 0.3 U) or saline (control). Additionally, naïve animals were included as needle-control. At P6, gray matter injury evaluated using MAP-2 immunohistochemistry showed that saline injection did not result in brain injury when compared to naïve controls. GMH-mediated injury could be detected 24 h following ICV injection, with significant injury in the 0.3 U collagenase group (Figure 1B). MAP-2 analysis showed a significant tissue reduction specifically within the ipsilateral striatum brain region when compared to naïve (*P* = 0.0050), saline- (*P* = 0.0381), and 0.1 U-injected (*P* = 0.0038) rats (Figure 1C).

Microglia showed significant ipsilateral morphological changes, changing from resting to focal activation in the 0.3 U group (Figures 1D,E). This activation was significant when compared to all other groups: naïve (*P* = 0.0008), 0.1 U (*P* = 0.0019), and 0.2 U (*P* = 0.0236) (Figure 1F). No increased astroglial immunoreactivity was detected in any of the different injection groups (Supplementary Figure 2A).

### Characterization of the Optimal Dose

**FIGURE 2:**
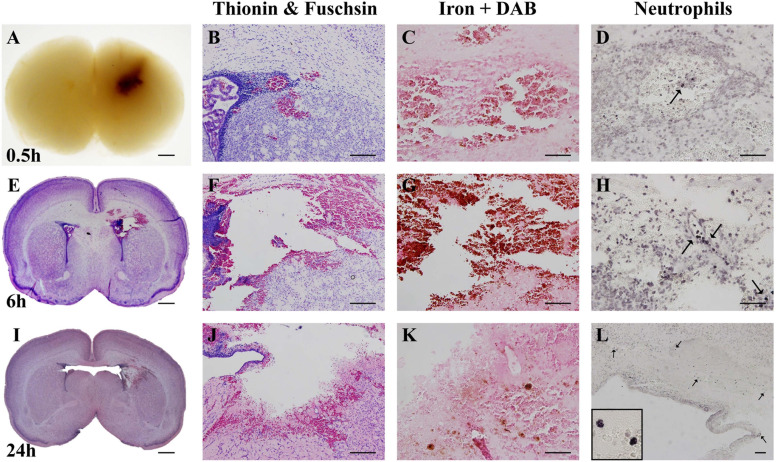
Characterization of optimal collagenase dose. **(A)** Coronal view of paraffin-embedded rat brain collected 0.5 h after 0.3 U collagenase injection. Representative micrograph of **(B)** thionin and fuchsin, **(C)** DAB-enhanced iron and **(D)** Neutrophil (MPO) stainings showing ipsilateral striatum 0.5 h after 0.3 U collagenase injection (*n* = 7). **(E)** Whole brain (thionin and fuschisin) and ipsilateral striatum level representative micrographs of **(F)** thionin and fuchsin, **(G)** DAB-enhanced iron and **(H)** Neutrophil (MPO) 6 h after 0.3 U collagenase injection (*n* = 7). **(I)** Whole brain (thionin and fuchsin) and ipsilateral striatum level representative micrographs of **(J)** thionin and fuchsin, **(K)** DAB-enhanced iron, and **(L)** Neutrophil (MPO) 24 h after 0.3 U collagenase injection (*n* = 12). Scale bar = 1 mm in **(A,E,I)**; 100 μm in all other micrographs. MPO, myeloperoxidase.

Both macroscopic and microscopic descriptive assessments showed that the injury obtained in the 0.3 U collagenase-injected animals resulted in near-immediate bleeding. This bleeding was visible macroscopically at 0.5 h after injection (Figure 2A). Microscopic observation at this time-point demonstrated localized presence of erythrocytes, as observed in the thionin and fuschsin stain (Figure 2B). DAB-enhanced iron staining showed clear presence of iron (Figure 2C), however, there was minimal infiltration of MPO-positive neutrophils (Figure 2D). By 6 h after collagenase injection, thionin and fuchsin-stained coronal sections showed clear tissue loss surrounding the injection site (Figure 2E), as well as structural disorganization in the penumbra (Figure 2F). This was associated with substantial iron increase (Figure 2G) and a small increase in neutrophil numbers located within the injury site (Figure 2H). Both tissue loss and structural disorganization increase over time, as seen 24 h after insult (Figure 2I), together with the presence of cells with pyknotic nucleus within the site of injury (Figure 2J). Interestingly, at this time-point, i.e., at P6, there was almost a complete clearance of iron at the site of injury (Figure 2K), as well as clear infiltration of neutrophils also in the surrounding parenchyma (Figure 2L).

**FIGURE 3:**
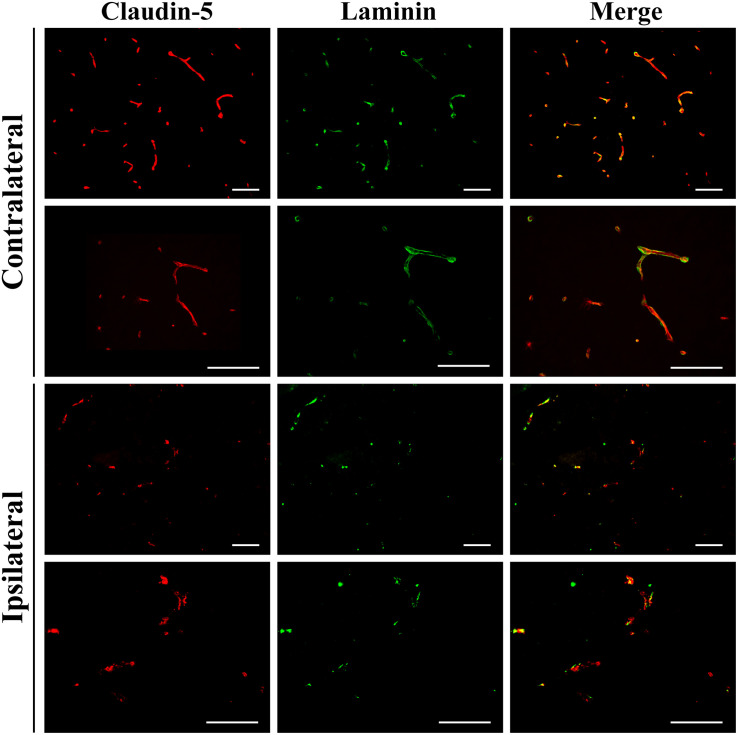
Vascular damage 24 h after 0.3 U collagenase infusion. Immunofluorescence microscopy demonstrating representative claudin-5 and laminin expression in intact vessels of the contralateral striatum at ×20 (top row) and ×40 (second row) magnifications 24 h after 0.3 U collagenase ICV infusion. Conversely, within the ipsilateral striatum there is clear vessel rupture with loss of both claudin-5 and particularly of laminin, as seen at ×20 (third row) and ×40 (fourth row) magnifications (*n* = 12). Scale bar = 100 μm.

To investigate damage to the basal lamina and the blood-brain barrier of cerebral blood vessels immunofluorescence was carried out for laminin and claudin-5 on brain sections at 24 h after GMH (Figure 3). This showed extensive loss of laminin around blood vessels in the striatum of the injured/ipsilateral hemisphere. Key blood-brain barrier protein claudin-5 appeared fragmented in blood vessels at the injury site compared to the uninjured hemisphere. In general, claudin-5 immunoreactivity was more visible on blood vessels than laminin as some blood vessels with claudin-5 immunoreactivity were devoid of laminin.

### Early Neurodevelopmental and Neuropathological Brain Injury Evaluation

**FIGURE 4:**
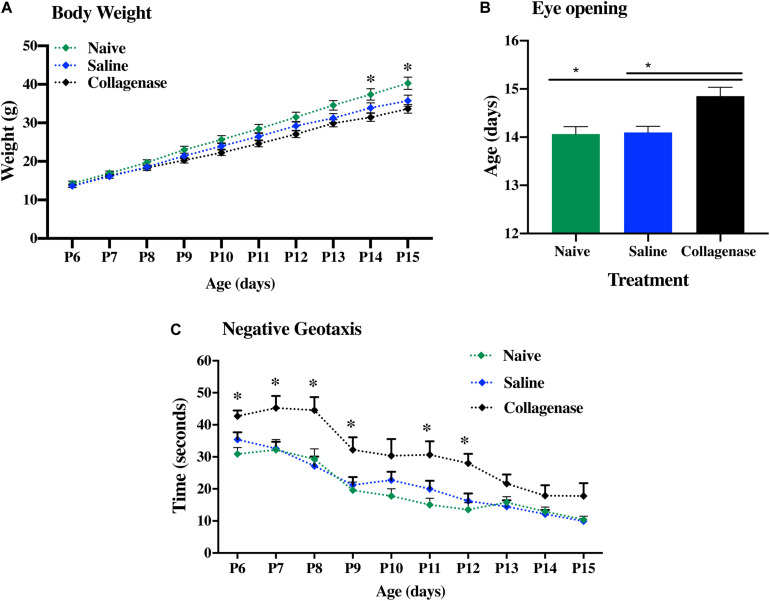
Neurodevelopmental deficits following GMH. **(A)** Weight gain over time, **(B)** Latency in eye opening, and **(C)** negative geotaxis assessment of naïve (*n* = 10), saline (*n* = 10), and 0.3 U collagenase (*n* = 12) animals. Data represented as mean ± SEM and analyzed using Kruskal-Wallis Dunn’s test. ^∗^*P* < 0.05–0.001.

Developmental assessment was performed in naïve (*n* = 10), saline- (*n* = 10), and collagenase-injected (*n* = 12) animals over a period of 10 continuous days, ranging between P6 and 15. The body weight of each pup was recorded daily. All three groups showed continued weight gain over time. However, at both P14 and P15, collagenase infused rats showed significantly less weight gain when compared to naïve animals (*P* = 0.0205 and *P* = 0.0277, respectively) (Figure 4A). By P14, around 80% of both naïve and saline-treated animals had opened their eyes, whereas in the collagenase group, only 45% had opened their eyes at this time point (*P* = 0.0205 and *P* = 0.0147, respectively). Furthermore, 33% of the pups in the collagenase group did not open their eyes until P16 whereas all animals in both naïve and saline groups had opened their eyes by P15 (Figure 4B). Negative geotaxis assessment demonstrated a significant impairment in the ability to rotate 180° upwards in the collagenase injected animals compared with both naïve and saline control groups at P6 (*P* = 0.0006 and *P* = 0.0325, respectively), P7 (*P* = 0.0706 and *P* = 0.0602, respectively), P8 (*P* = 0.0270 and *P* = 0.0602, respectively), naïve only at P9 (*P* = 0.0317) and P11 (*P* = 0.0317), and again both naïve and saline groups at P12 (*P* = 0.0024 and *P* = 0.0425, respectively) (Figure 4C).

**FIGURE 5:**
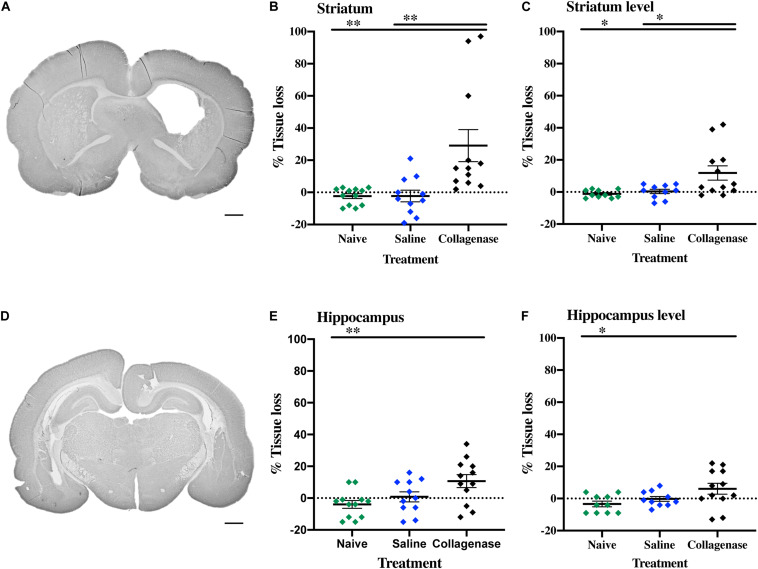
Gray matter injury at P16. **(A)** Representative MAP-2-stained whole brain micrograph of 0.3 U collagenase-mediated striatal injury at P16. **(B)** Graph demonstrating striatum-specific tissue loss and **(C)** striatum level ipsilateral hemisphere loss in the collagenase group. **(D)** Representative MAP-2-stained whole brain micrograph of 0.3 U collagenase-mediated hippocampal injury at P16. **(E)** Graph demonstrating hippocampus-specific tissue loss and **(F)** hippocampus level ipsilateral hemisphere loss in the collagenase group (*n* = 12). Both naïve (*n* = 10) and saline-injected (*n* = 10) animals were used as control groups. Data represented as individual animals ± SEM and analyzed using one-way ANOVA followed by Tukey’s multiple comparison test. ^∗^*P* < 0.05 and ^∗∗^*P* < 0.01. Scale bar = 1 mm.

At P16, the injury found in the collagenase group consisted of asymmetrical lateral ventricles with slight to severe ipsilateral dilation associated with shrinkage of the striatum, and occasionally, of the hippocampus. MAP-2 assessment showed significant reduction of gray matter in the striatum (Figure 5A) and hippocampus (Figure 5D) regions in the collagenase group: striatum naïve (*P* = 0.00031), striatum saline (*P* = 0.0039) (Figure 5B), and hippocampus naïve (*P* = 0.0090) (Figure 5E).

By this time-point, there was a progression in injury extent, resulting in significant gray matter loss when assessing entire hemispheres, with a significant reduction in positive MAP-2 at the ipsilateral hemisphere at the striatum level when compared to naïve (*P* = 0.0118) and saline (*P* = 0.0275) controls (Figure 5C), and hippocampus level when compared to naïve animals (*P* = 0.0320) (Figure 5F).

**FIGURE 6:**
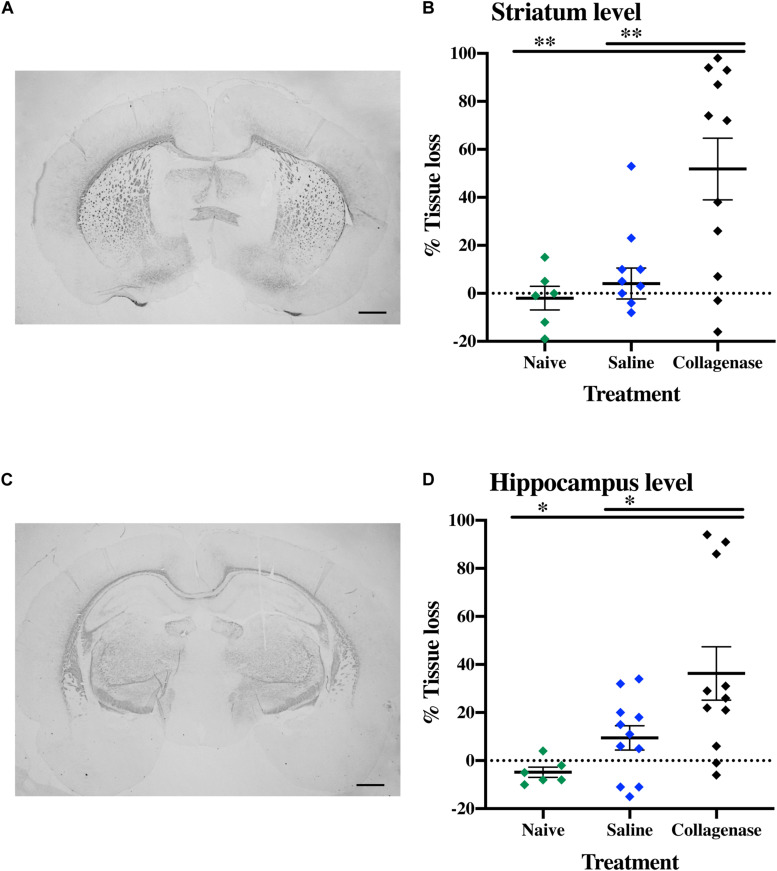
White matter injury at P16. **(A)** Representative striatum level MBP-stained whole brain micrograph of 0.3 U collagenase-injected rats and **(B)** corresponding graph demonstrating collagenase-mediated reduction in subcortical white matter at P16. **(C)** Representative MBP-stained whole brain micrograph of 0.3 U collagenase-injected rats at the level of the hippocampus and **(D)** associated graph showing subcortical white matter in the collagenase group at P16 (*n* = 12). Both naïve (*n* = 10) and saline (*n* = 10) animals were used as control groups. Data represented as individual animals ± SEM and analyzed using one-way ANOVA followed by Tukey’s multiple comparison test. ^∗^*P* < 0.05 and ^∗∗^*P* < 0.01. Scale bar = 1 mm.

MBP assessment showed a significant reduction in subcortical white matter in the collagenase group, both at the striatum (Figure 6A) and hippocampus (Figure 6C) levels when compared to naïve (*P* = 0.0054 and *P* = 0.0111, respectively) and saline controls (*P* = 0.0034 and *P* = 0.0542, respectively (Figures 6B,D).

**FIGURE 7:**
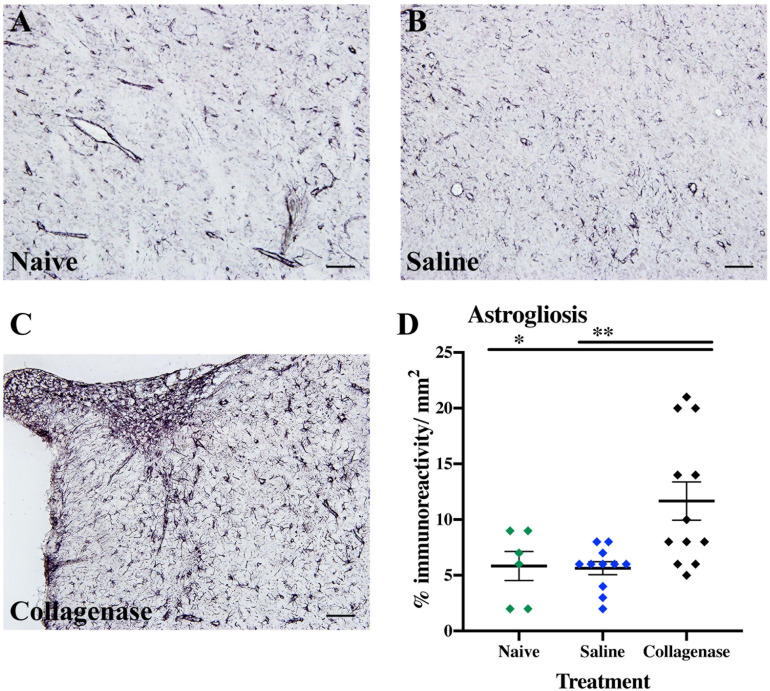
GMH induces astrogliosis at P16. **(A)** Representative micrograph of GFAP staining in the ipsilateral striatum of naïve (*n* = 10), **(B)** saline (*n* = 10), and **(C)** collagenase (*n* = 12) groups at P16. **(D)** Corresponding graph showing increase in GFAP immunoreactivity in the collagenase-injected group at P16. Data represented as individual animals ± SEM and analyzed using one-way ANOVA followed by Tukey’s multiple comparison test. ^∗^*P* < 0.05 and ^∗∗^*P* < 0.01. Scale bar = 100 μm.

IBA-1 measurements showed no microglia activation in the collagenase-injected animals at this time-point (Supplementary Figure 2B). However, astrogliosis was significantly present at the striatum level, when compared to both naïve (*P* = 0.0294) and saline-injected (*P* = 0.0063) animals (Figures 7A–D).

### Effect of GMH on Long-Term Sensorimotor and Cognitive Functions

**FIGURE 8:**
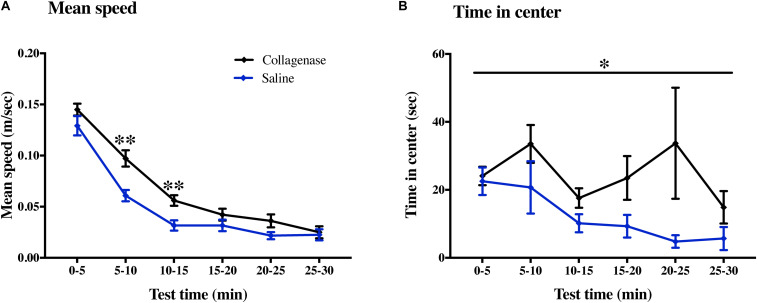
Effect of GMH on long-term sensorimotor and cognitive functions. At P40, the open field test showed that **(A)** mean speed and **(B)** time spent in the center was affected by GMH (*n* = 19) when compared to saline controls (*n* = 12). Data represented as mean ± SEM and analyzed using two-way mixed ANOVA. ^∗^*P* < 0.05 and ^∗∗^*P* < 0.01.

Sensorimotor and cognitive functions were assessed twice using a series of tests between P22–26 and P36–40. On P37, collagenase infused animals moved faster compared with the control animals in 5–10 min (*P* < 0.001) and 10–15 min (*P* < 0.001) (Figure 8A) time bins of the open field test. Collagenase-injected animals spent significantly longer time in the center of the open field compared with the control animals throughout the test (*P* < 0.05) (Figure 8B). There was no difference between the collagenase and control animals in all other behavior tests at either of the time points (Supplementary Figure 3). There was no difference between the saline-injected and naïve animals in any of the behavior tests.

**FIGURE 9:**
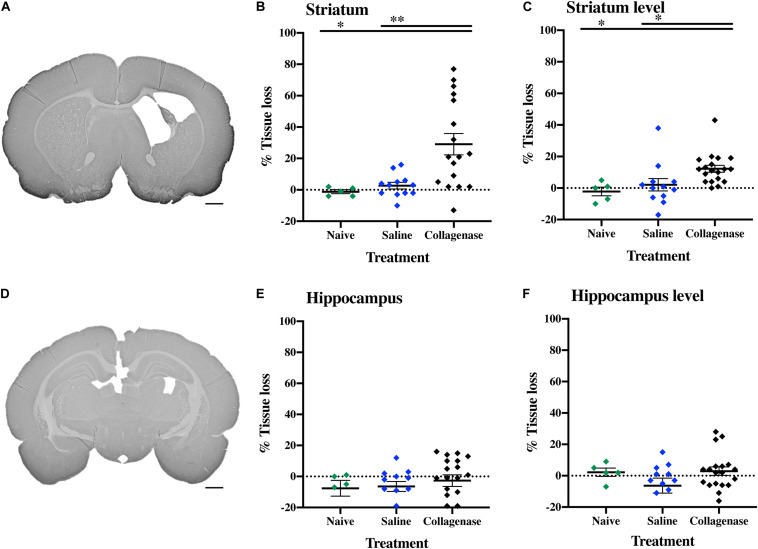
Long term gray matter injury. **(A)** Representative MAP-2-stained whole brain micrograph of 0.3 U collagenase-mediated striatal injury at P40. **(B)** Graph demonstrating striatum-specific tissue loss and **(C)** striatum level ipsilateral hemisphere loss in the collagenase group. **(D)** Representative MAP-2-stained whole brain micrograph of 0.3 U collagenase-injected rat at the hippocampal level at P40. **(E)** Corresponding graph revealing non-significant hippocampus-specific tissue loss and **(F)** hippocampus level ipsilateral hemisphere measurement in the collagenase group (*n* = 19). Both naïve (*n* = 5) and saline-injected (*n* = 12) animals were used as control groups. Data represented as individual animals ± SEM and analyzed using one-way ANOVA followed by Tukey’s multiple comparison test. ^∗^*P* < 0.05 and ^∗∗^*P* < 0.01. Scale bar = 1 mm.

**FIGURE 10:**
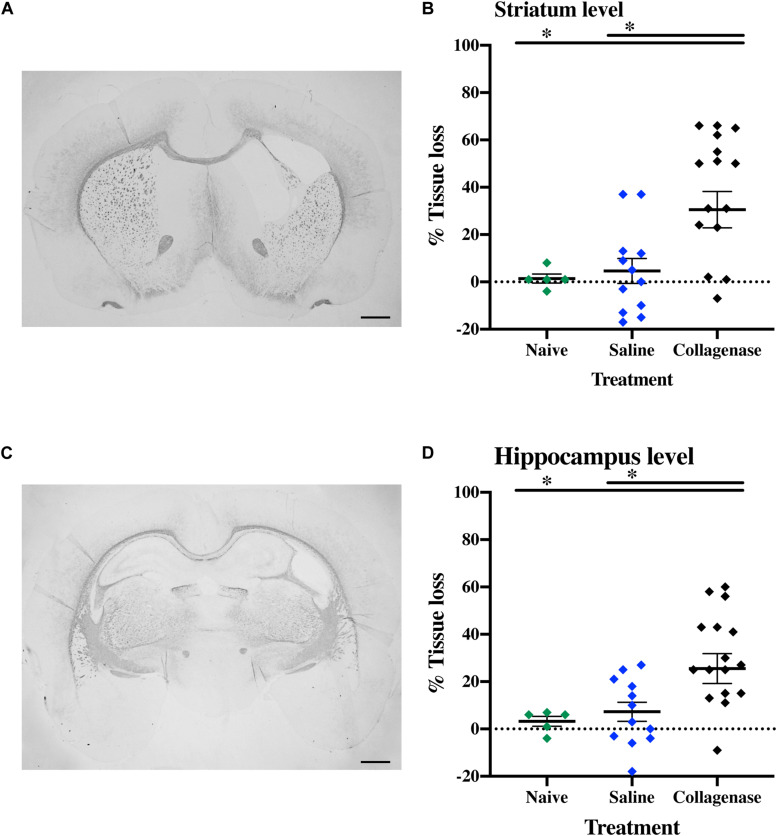
Long term white matter injury at P40. **(A)** Representative MBP-stained whole brain micrograph of 0.3 U collagenase-injected rats at the level of the striatum and **(B)** corresponding graph demonstrating collagenase-mediated reduction in subcortical white matter at P40. **(C)** Representative MBP-stained whole brain micrograph of 0.3 U collagenase-injected rats at the level of the hippocampus and **(D)** associated graph showing subcortical white matter in the collagenase group (*n* = 19) at P40. Both naïve (*n* = 5) and saline (*n* = 12) animals were used as control groups. Data represented as individual animals ± SEM and analyzed using one-way ANOVA followed by Tukey’s multiple comparison test. ^∗^*P* < 0.05. Scale bar = 1 mm.

In brains from animals that had previously been assessed for behavior there was significant gray matter tissue loss within the striatum region (Figure 9A) of collagenase animals (*n* = 19) when compared to the naïve (*n* = 5) (*P* = 0.0189) and saline (*n* = 12) (*P* = 0.0053) groups (Figure 9B). Tissue loss was also significant in terms of total hemisphere loss (*P* = 0.0325 and *P* = 0.0420, respectively) (Figure 9C). There was no significant gray matter tissue loss at the hippocampus level (Figures 9D–F). MBP assessment at the striatum level (Figure 10A) showed significant reduction in subcortical white matter in the collagenase group when compared to naïve (*P* = 0.0452) and saline controls (*P* = 0.0273) (Figure 10B). At the hippocampus level, collagenase injection resulted in significant reduction in MBP (Figure 10C) when compared to naïve (*P* = 0.0482) and saline-injected animals (*P* = 0.0431) (Figure 10D). There was no significant astrogliosis or microglia activation in the collagenase group at this time-point (Supplementary Figures 4A,B).

## Discussion

In the current study we introduce a P5 rat model of GMH using intracranial striatal collagenase injections in proximity to the germinal matrix. Collagenase administration results in a standardized rupture of the vessels in the germinal matrix of the ganglionic eminence, associated with bleeding into the lateral ventricle of the injected hemisphere and reduction in white matter surrounding both the striatum and the affected ventricle.

Lekic et al. (2011, 2012) were the first group using unilateral 0.3 U collagenase VII injection into the germinal matrix of P7 rats mimicking pathological features present in clinical cases of GMH-IVH. However, in these studies, the collagenase infusion resulted in bilateral IVH (Lekic et al., 2011, 2012), which only occurs in a minority of cases at P5. A study by Maitre et al. (2009) has shown that unilateral GMH-IVH occurred in 75% of clinical cases, with bilateral cases consisting of only 15%. Furthermore, recent studies have suggested that in terms of brain maturation, P7 age in rats is closer to near-term human infants (Semple et al., 2013). A gestational age where the germinal matrix is no longer present and GMH is no longer considered to be one of the main risk factors for neonatal brain injury. Therefore, we sought to adapt this model to the P5 rat, which is comparable to that of a human fetus or newborn at 26–32 gestational weeks with respect to cortical developmental stages (Honig et al., 1996), presence of germinal matrix and maturation of white matter (Back et al., 2001).

Firstly, we sought to determine a collagenase infusion dose that would mimic the pathophysiology seen in clinical cases. Among the three different doses of collagenase VII (0.1, 0.2, and 0.3 U), only 0.3 U of collagenase resulted in ipsilateral striatal injury, as well as microglial activation surrounding the site of injury 24 h after injection. Clinically, microglia immune-response has been implicated in preterm brain injury, and have been shown to accumulate in the periventricular region in the first 48 h after IVH (Supramaniam et al., 2013; Mallard et al., 2014).

Further characterization of this dose showed a clear acute injury. Histological assessment 0.5 h after 0.3 U collagenase administration resulted in immediate bleeding within the injection site, as well as within the lateral ventricle and the appearance of iron. Within 6 h, there was a substantial increase in erythrocytes and deposits of loosely bound iron that also extended to the white matter surrounding the ipsilateral ventricle, together with focal infiltration of neutrophils. By 24 h there seemed to be an erythrocyte and iron clearance, whereas neutrophils appeared to migrate to the surrounding parenchyma. This neutrophil infiltration is in concordance with both clinical and preclinical GMH studies. Paul et al. (2000) found that peripheral leukocytes and absolute neutrophils were increased in the first 72 h after birth in extremely preterm IVH cases. In a rabbit model of GMH-IVH, there was a greater number of microglia and neutrophils in the periventricular zone compared to control animals (Georgiadis et al., 2008).

In preterm infants, the site of origin of bleeding generally occurs in small blood vessels of the germinal matrix. GMH can result in disruption of the ependymal lining and extend the bleeding into the lateral ventricle. Germinal matrix vessels are fragile and have an endothelial layer with underdeveloped tight junctions, small pericyte numbers, and fibronectin deficiency in the immature basal lamina. Astrocyte end-feet have lower levels of glial fibrillary acidic protein expression. All these contribute to the rupture of germinal matrix blood vessels, and potential subsequent extravasation of blood into the lateral ventricle (Ballabh et al., 2004). We have shown extensive loss of laminin of blood vessels within the ipsilateral striatum 24 h after collagenase infusion, with some vessels devoid of laminin, indicating extensive damage to the basal lamina. Claudin-5, a key component of the blood-brain barrier also appeared fragmented in the vessels surrounding the site of injury.

Post hemorrhagic hydrocephalus is a clinical feature of GMH-IVH. It is thought that ventricular dilatation probably ensues soon after the hemorrhage in many preterm infants and is associated with poor prognosis (Brouwer et al., 2014). In the current study, the collagenase injection induced a periventricular/intraventricular hemorrhage best visualized 24 h after injection. The hemorrhage resulted in a progressive ipsilateral ventricular dilation at later time points, combined with continual loss of gray and white matter surrounding the site of injection.

Despite increase in survival of preterm infants, neurodevelopmental delay is a hallmark of GMH cases (Pierrat et al., 2017). We also found a quite marked initial deficit in motor coordination detected in the negative geotaxis test that persisted 1 week after collagenase admnistration. Interestingly, this developmental deficit lasted longer that observed by Lekic et al. (2012) which only saw significant differences in the first 48 h post-GMH (Alles et al., 2010). The successful completion of this task requires organized motor movement (Adams et al., 1985) and the initial abnormal outcome in this test indicates that the hemorrhage induced by intracranial striatal collagenase injection strongly affects development of the motor coordination during this period and seems to be a robust test for evaluation of early motor outcome in this model.

All animals were observed daily, and the body weight of each pup was recorded daily during this period. Despite a continual weight gain in all groups, by P14 collagenase infused rats had gained less weight than naïve animals. Coincidently, this is the age period (P14–15) when the day of eyelid opening occurs in rats (Bjerknes et al., 2015). This constitutes the starting point of the rats’ exploratory behavior outside of the nest (Langston et al., 2010). We observed that animals exposed to GMH-IVH opened their eyes significantly later than naïve and saline-injected animals. These findings are in agreement with the results by Lekic et al. (2011, 2012) P7 study. Histological assessment of these animals revealed development of ventricular dilation, together with significant gray and white matter injury. Additionally, GFAP immunohistochemistry measurement showed significant astrogliosis within the ipsilateral striatum region.

Clinically, MRI is a tool commonly used to predict short- and long-term neurodevelopmental outcomes. The presence of white matter injury, in the form of delayed myelination, white matter loss, ventriculomegaly, among others have shown a strong correlation with cerebral palsy and other neuromotor deficits (Volpe, 2009; Anderson et al., 2015; van’t Hooft et al., 2015; Slaughter et al., 2016). We tested long-term sensorimotor and cognitive functions in a separate group of juvenile rats 2–3 weeks (P22–26) and 4–5 weeks (P36–40) after collagenase infusion. There was a significant increase in hyperactivity and a decrease in anxiety-like behavior in collagenase infused animals on P37 compared with the control group. However, motor coordination and motor asymmetry remained intact in the collagenase infused animals at both time points. Alles et al. (2010) reported similar findings in a P6 rat model of PVH/IVH using a much higher dose of collagenase (2.0 U), suggesting a compensatory neuroplasticity originating from the intact contralateral hemisphere. Subsequently, a bilateral infusion of collagenase caused significant impairments in both sensorimotor and cognitive functions that was apparent at the juvenile stage (Alles et al., 2010). Conversely, Lekic et al. reported a significant impairment in long-term sensorimotor and cognitive behavior in rats injected with the same dose of collagenase as used in the current study. However, unlike the current study, the authors observed a bilateral ventricular dilation and brain atrophy. Omizzolo et al. (2014) have reported that injury to the gray matter areas of the basal ganglia-thalamus of very preterm infants showed the strongest prediction for memory and learning outcome at 7 years of age. Infants with unilateral PVH infarction are found to have better sensorimotor/cognitive outcomes and are less likely to develop severe cerebral palsy compared to infants with bilateral PVH injury (Maitre et al., 2009). Gray matter histological assessment showed continued striatal injury with an almost unimpaired hippocampus in the ipsilateral hemisphere. The consistent pattern of injury in striatum suggests that in future studies should include striatum-dependent cognitive tasks, such as operant conditioning. MBP measurement of subcortical white matter thickness revealed a persistent long-term white matter injury around the fluid-filled ventricle.

Animal models are frequently used to study injury progression and pathophysiological mechanisms involved. In this study we have successfully shown that injection of collagenase VII into the medial striatum in proximity to the germinal matrix of the preterm equivalent P5 rat results in acute injury, leading to progressive ventricular dilation and secondary gray and white matter injury associated with impaired motor function during early development. Gray and white matter injury persisted into the juvenile period combined with hyperactivity and reduced anxiety. Overall, our model has relevance for mimicking GMH in preterm infant and has potential for assessment of therapeutic interventions.

## Data Availability Statement

The datasets generated for this study are available on request to the corresponding author.

## Ethics Statement

The animal study was reviewed and approved by the Swedish Board of Agriculture and were approved by the Gothenburg Animal Ethics Committee (825-2017).

## Author Contributions

MJ and GK performed the animal experiments and developmental testing, and together with A-LL and PS carried out tissue preparation and processing. AJ and ER-F carried out the immunohistological analyses. CE carried out immunofluorescence analysis and SN assisted in the processing of all images and figures. GS-M and MJ performed behavioral testing and analysis. XW and CM participated in data interpretation. ST contributed to the funding and participated in the planning of the study. ER-F designed experiments, performed statistical analysis, interpreted data, and wrote the manuscript together with GK and HH. HH conceptualized and designed the study, assisted in data interpretation, and obtained funding. All authors reviewed and revised the manuscript and approved the final manuscript as submitted.

## Conflict of Interest

The authors declare that the research was conducted in the absence of any commercial or financial relationships that could be construed as a potential conflict of interest.

## Supplementary Material

Supplementary Figure 1 Schematic diagram of the different treatment groups, time-points, and assessments performed across the study.

Supplementary Figure 2 **(A)** Assessment of astrogliosis (GFAP) in the ipsilateral striatum 24 h after different collagenase dose administrations, with groups consisting of: naïve (*n* = 8), saline (*n* = 8), 0.1 U (*n* = 8), 0.2 U (*n* = 9), and 0.3 U (*n* = 9) collagenase administration. **(B)** Assessment of microglia activation (IBA-1) in the ipsilateral striatum at P16 of naïve (*n* = 10), saline (*n* = 10), and 0.3 U collagenase (*n* = 12) animals. Data represented as individual animals ± SEM and analyzed using one-way ANOVA followed by Tukey’s multiple comparison test.

Supplementary Figure 3 Data from rotarod at **(A)** P26 and **(B)** P40, cylinder rearing test at **(C)** P26 and **(D)** P40 and P23 novel object recognition **(E)** mean speed and **(F)** time in the center. Collagenase injection (*n* = 19) did not affect behavioral function when compared to saline-injected (*n* = 12). Data represented as mean ± SEM and analyzed using two-way mixed ANOVA or unpaired *t*-test.

Supplementary Figure 4 **(A)** Assessment of astrogliosis (GFAP) and **(B)** microglia activation (IBA-1) at P40 between naïve (*n* = 5), saline (*n* = 12), and collagenase (*n* = 19) groups. Data represented as individual animals ± SEM and analyzed using one-way ANOVA followed by Tukey’s multiple comparison test.
