# Supplementary figures and images for: A Model of Germinal Matrix Hemorrhage in Preterm Rat Pups
Source: Front Cell Neurosci. 2020 Dec 3;14:535320. doi: 10.3389/fncel.2020.535320 (PMC7744792; doi:10.3389/fncel.2020.535320)

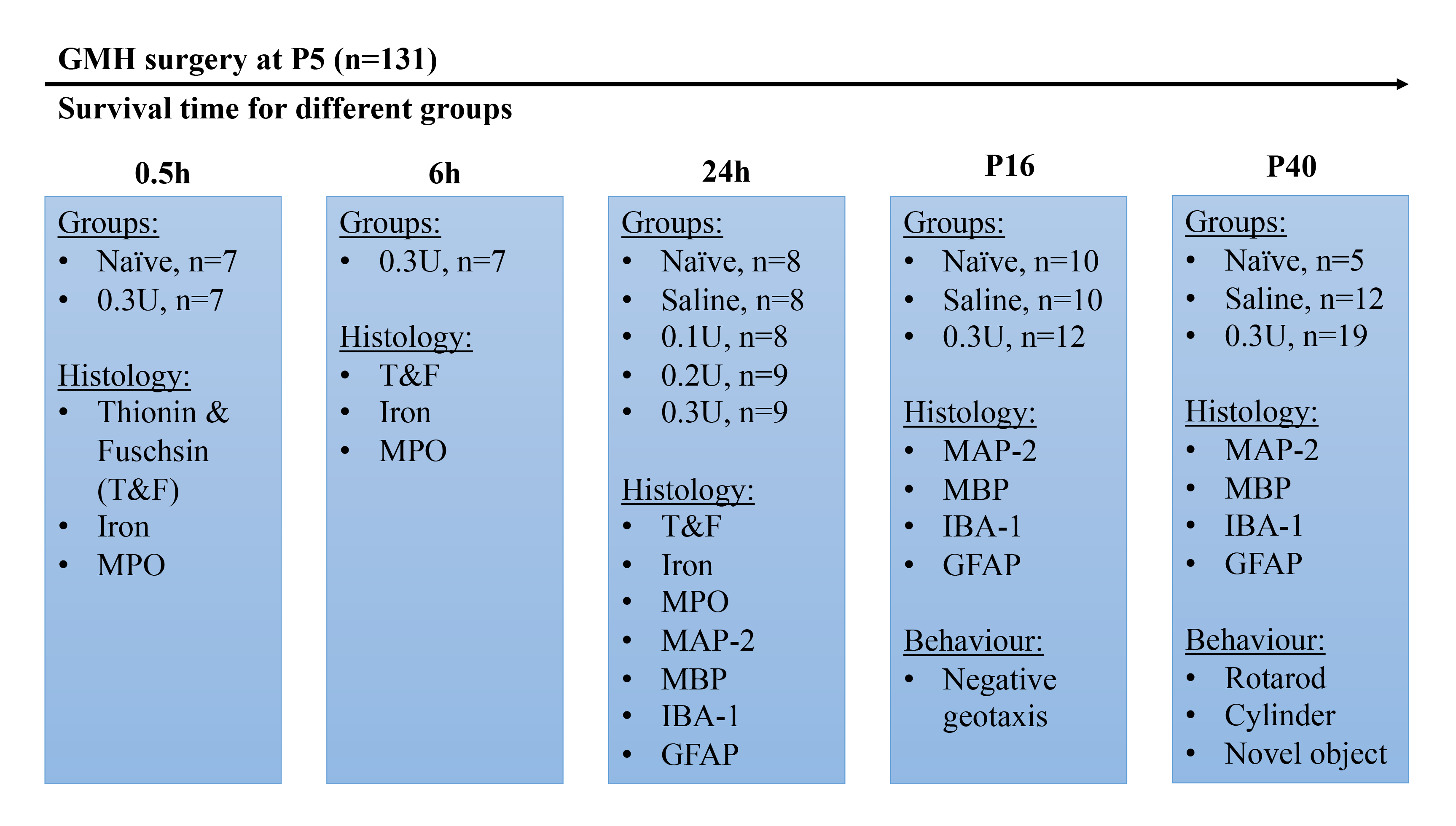

Supplement: Supplementary Figure 1 — Schematic diagram of the different treatment groups, time-points, and assessments performed across the study. [file Image_1.TIF]

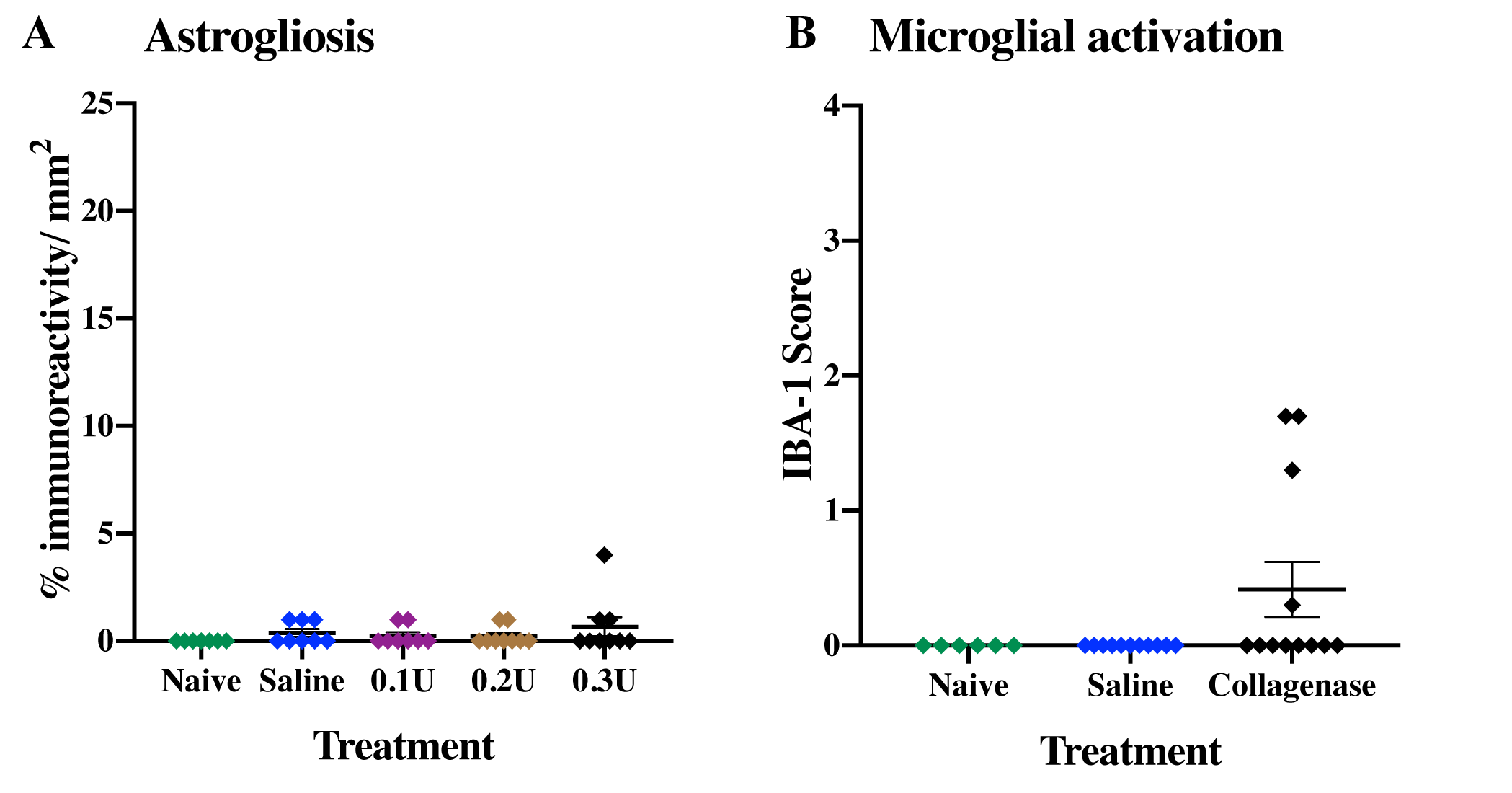

Supplement: Supplementary Figure 2 — (A) Assessment of astrogliosis (GFAP) in the ipsilateral striatum 24 h after different collagenase dose administrations, with groups consisting of: naïve (n = 8), saline (n = 8), 0.1 U (n = 8), 0.2 U (n = 9), and 0.3 U (n = 9) collagenase administration. (B) Assessment of microglia activation (IBA-1) in the ipsilateral striatum at P16 of naïve (n = 10), saline (n = 10), and 0.3 U collagenase (n = 12) animals. Data represented as individual animals ± SEM and analyzed using one-way ANOVA followed by Tukey’s multiple comparison test. [file Image_2.TIF]

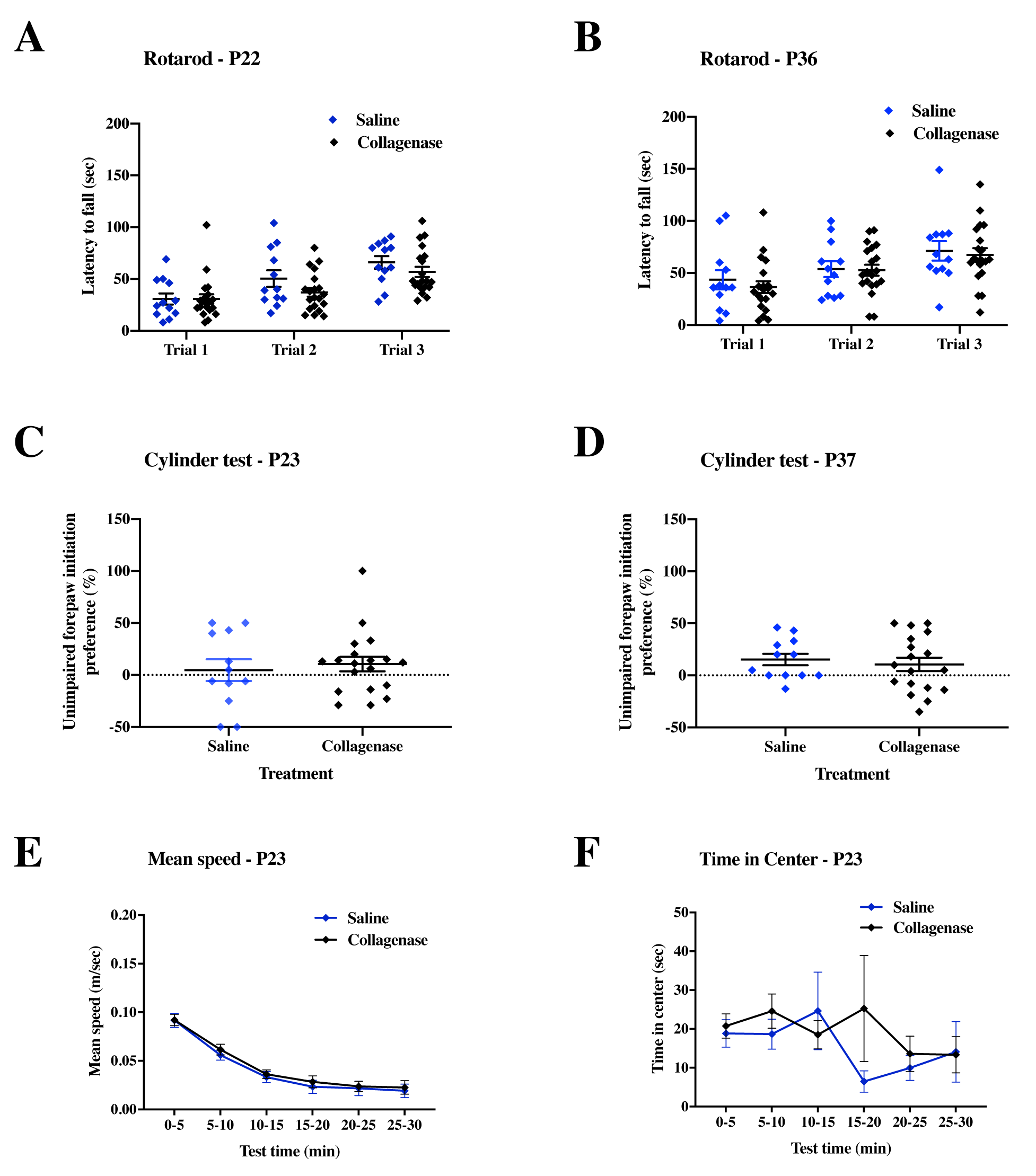

Supplement: Supplementary Figure 3 — Data from rotarod at (A) P26 and (B) P40, cylinder rearing test at (C) P26 and (D) P40 and P23 novel object recognition (E) mean speed and (F) time in the center. Collagenase injection (n = 19) did not affect behavioral function when compared to saline-injected (n = 12). Data represented as mean ± SEM and analyzed using two-way mixed ANOVA or unpaired t-test. [file Image_3.TIF]

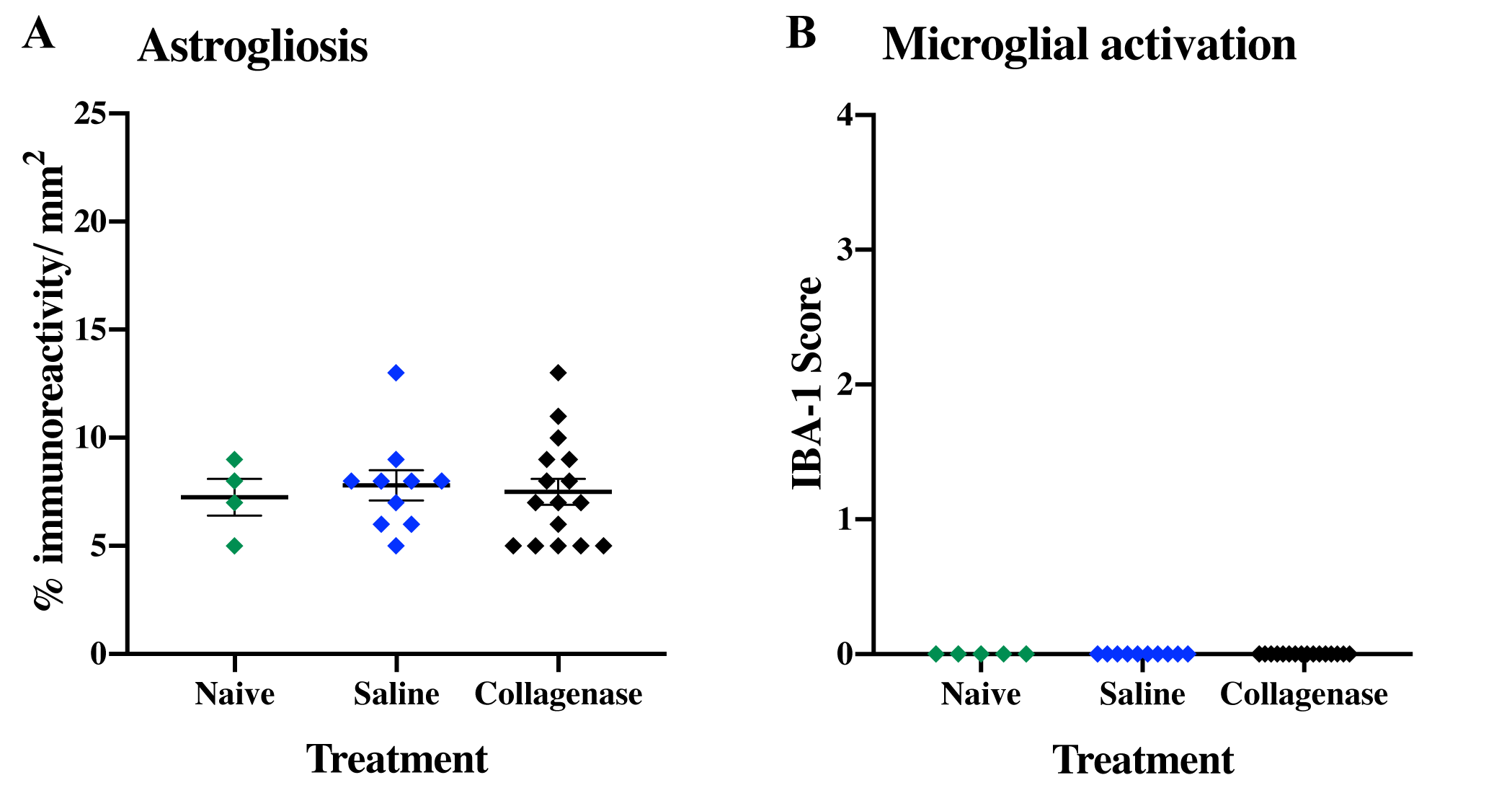

Supplement: Supplementary Figure 4 — (A) Assessment of astrogliosis (GFAP) and (B) microglia activation (IBA-1) at P40 between naïve (n = 5), saline (n = 12), and collagenase (n = 19) groups. Data represented as individual animals ± SEM and analyzed using one-way ANOVA followed by Tukey’s multiple comparison test. [file Image_4.TIF]
